# Primary myoepithelial carcinoma of palate

**DOI:** 10.1186/1477-7819-9-104

**Published:** 2011-09-14

**Authors:** Juan Ren, Zi Liu, Xiaoping Liu, Yi Li, Xiaozhi Zhang, Zongfang Li, Yunyi Yang, Ya Yang, Yuanyuan Chen, Shiwen Jiang

**Affiliations:** 1 Cancer center, First Hospital of Xi'an Jiaotong University, Xi'an 710061, Shaan'xi Province, 710061, China; 2 Department of Basic Biomedical Sciences, Mercer University School of Medicine, GA 31404, USA; Department of Obstetrics and Gynecology, Mayo Clinic, Rochester, MN 55905, USA; 3 Second Hospital of Xi'an Jiaotong University, Xi'an 710061, Shaan'xi Province, 710061, China

**Keywords:** Myoepithelial carcinoma, Palate, Myoepitheliomas

## Abstract

**Objectives:**

The aim of this study was to present a rare neoplasm, Primary myoepithelial carcinoma arising from the palate, and to review its diagnostic criteria, pathologic and clinical characteristics, treatment options and prognosis.

**Clinical Presentation and Intervention:**

Myoepitheliomas are tumors arising from myoepithelial cells mainly or exclusively. Myoepitheliomas mostly occur in salivary glands, as well as in breast, skin, and lung. Case of myoepitheliomas in palate has rarely been reported. Myoepithelial carcinoma is malignant counterpart of myoepitheliomas. Adenomyoepithelioma is also a different disease from myoepitheliaomas. Immunohistochemically, tumor cells of myoepithelial carcinoma express not only epithelial markers such as cytokeratin, epithelial membrane antigen (EMA), but also markers of smooth muscle origin such as calponin. The immunohistochemical criteria of myoepithelial differentiation are double positive for both cytokeratins and one or more myoepithelial immunomarkers (i.e., S-100 protein, calponin, p63, GFAP, maspin, and actins). Myoepithelial carcinomas of salivary and breast demonstrate copy number gains and gene deletion. The overall prognosis of myoepithelial carcinoma is poor. There is rarely recurrence or metastasis in benign myoepithelial tumors. Complete excision with tumor-free margin is always the preferred treatment, while local radiation therapy and chemotherapy are suggestive treatment options. Here, a rare case of myoepithelial carcinoma arising from the palate has been described and discussed for the treatment and outcome. Pathological and clinical characters of myoepitheliomas are also compared and discussed.

**Conclusion:**

The case report serves to increase awareness and improve the index of diagnosis and treatment of myoepitheliomas.

## 1. Background

Myoepitheliomas are tumors arising from myoepithelial cells lacking ductal differentiation which exhibit both epithelial and smooth muscle cell characteristics. Benign myoepithelial tumors were seen mostly in extremities and head-neck region, while malignant counterparts mostly occur in the salivary gland, parotid, ans breast tissues.

Histopathology and immunohistochemistry play critical roles in diagnosis of myoepithelial carcinoma due to its differentiation limited to myoepithelium frequently. Myoepithelioma shows solid, reticular and trabecular arrangement histopathologically and is composed of round/epithelioid or spindle cells, frequently infiltrated by clear or plasmacytoid cells. Immunohistochemistry shows general positive for both epithelial and myogenic markers in myoepithelial carcinoma cells.

The most common arising sites of myoepithelial carcinoma lie in the parotid gland [1], as well as the nasopharynx, paranasal sinus and nasal cavity of head-neck region [2,3]. Myoepithelial carcinoma rarely occurs in the palate so the description is not adequate. Here we report a rare case of myoepithelial carcinoma arising from palate and describe its surgical and radiotherapy management and prognosis. Features of myoepitheliomas are also discussed through review the previous literature.

## 2. Case presentation

### 2.1 clinical reviews

The patient was a 75-year-old male, and there were unremarkable medical records for him and his family members. One year ago an unconscious eminentia was found in his right palate. There was no pain, discomfort or difficulties in eating or swallowing. The eminentia grew gradually and at last turned to a hard, ill-defined, oval mass with the diameter of 2.2 cm in the right palate, showing a blue black fleck on its surface membrane. There was no ulceration or mucosal erosion on the mass which demonstrated immovable and tenderness. Neither history of known malignant diseases, nor neoplasms by full-body imaging was found in this patient.

### 2.2 Pathological and immunohistochemical findings

**Figure 1:**
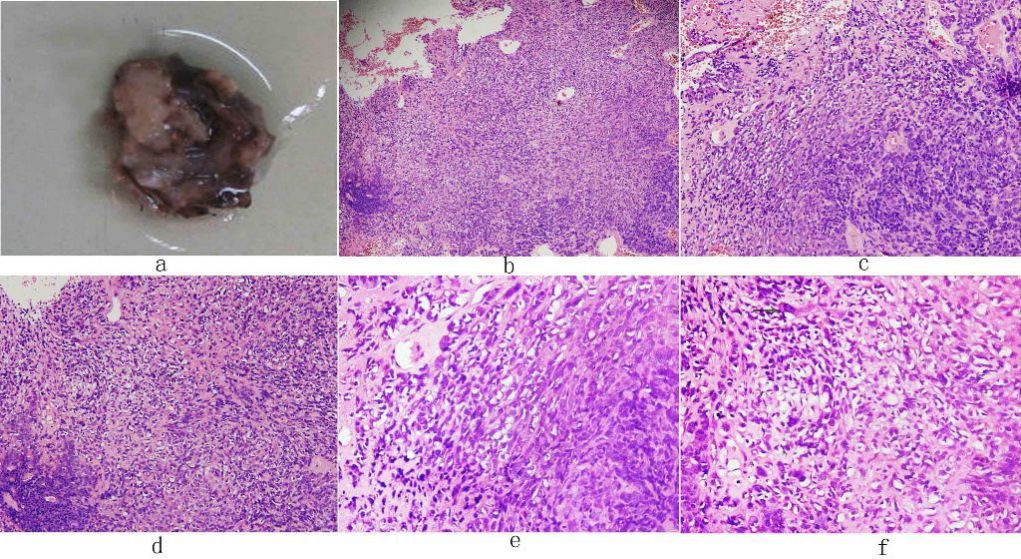
**HE staining**. (b): 10 ×; (C), (d): 20 ×; (e),(f): 40 ×.

Histological examinations were performed after surgery to confirm the diagnosis. The tumor showed a 22.52 × 25 mm, lobulated neoplasm with an off-white coarser surface gross appearance. Colors from red to gray were seen in a vertical section of the tumor. Through ultrastructural examination, the tumor cells showed macronuclei, multiple nuclei, increased mitosis, high density nuclear chromatin, and the tumor cell nuclei were circular with abundant euchromatin and a conspicuous nucleolus. Cubic cells (Figure 1a) and local cystic degeneration and hemorrhage were observed in the tumor, and were arranged in film (Figure 1b) and deeply stained in nucleus. The size of the nucleus varied from cell to cell. Some tumor cells showed translucent cytoplasm and tubular structure (Figure 1c). Cells showed oval and different sizes (Figure 1d) and were ill-defined (Figure 1e). Mitotic figure and delicate chromatin was found in nucleus (Figure 1f). Vacuolated cytoplasm and translucent cytoplasm were observed.

**Figure 2:**
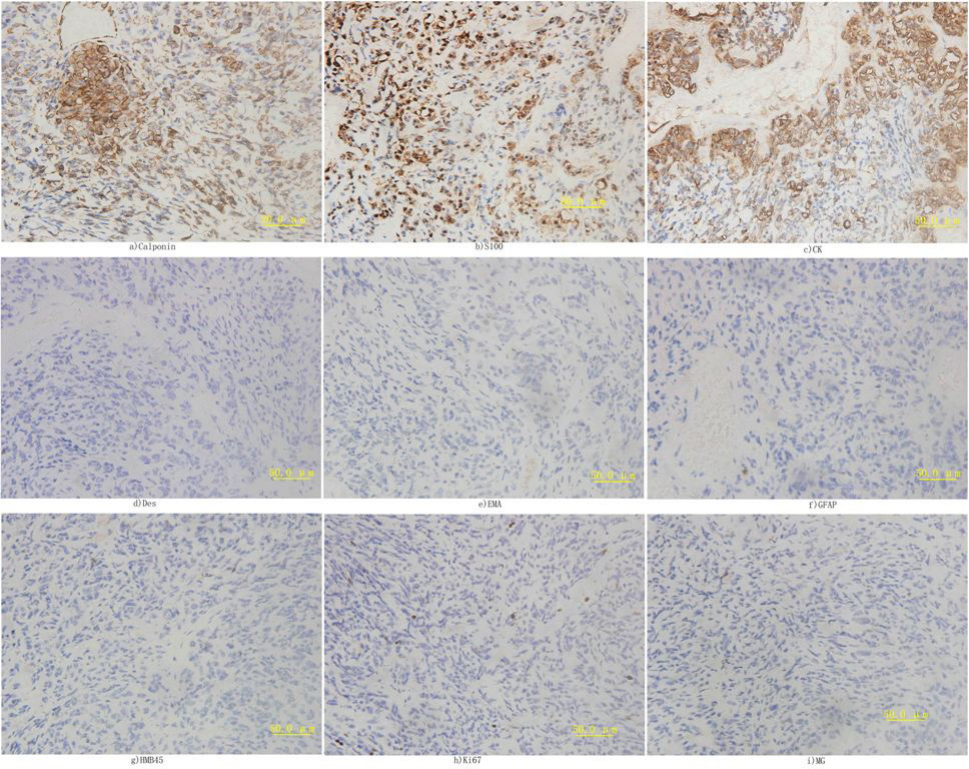
**Immunohistochemical staining, Brown particle was regarded as positive staining signal**. (a), Immunostaining of Calponin (200 ×), Calponin antibody was from Zhong Shan and was 1:100 diluted; (b), Immunostaining of S-100 (200 ×), S-100 antibody was from Zhong Shan and was 1:80 diluted; (c), Immunostaining of cytokeratin (200 ×), CK antibody was from MaiXin and was 1:60 diluted; (d), Immunostaining of desmin (200 ×), desmin antibody was from Zhong Shan and was 1:80 diluted; (e), Immunostaining of EMA (200 ×), EMA antibody was from Zhong Shan and was 1:80 diluted; (f), Immunostaining of GFAP (200 ×), GFAP antibody was from MaiXin and was 1:80 diluted; (g), Immunostaining of human melanoma black45(HMB45) (200 ×), HMB45 antibody was from MaiXin and was 1:80 diluted; (h), Immunostaining of Ki-67 (200 ×), Ki-67 antibody was from Zhong Shan and was 1:80 diluted; (i), Immunostaining of microglobulin (200 ×), microglobulin antibody was from Zhong Shan and was 1:80 diluted.

Immunohistochemical staining showed the tumor was strongly positive for Cytokeratin, S-100, and Calponin, and was negative for epithelial membrane antigen (EMA), glial fibrillary acidic protein (GFAP), human melanoma black45 (HMB45), desmin, and microglobulin (Figure 2). Calponin and S-100 were highly expressed in the cytoplasm of myoepithelial cells, while Cytokeratin was expressed in the nucleus (in Figure 2a, b, c); Cytokeratin is expressed in both gland duct epithelial cells and myoepithelial cells. In our case, cytokeratin is positively stained in nucleus of myoepithelial cells (Figure 2c) which is consistent with the major diagnostic criteria of myoepithelial carcinoma.

### 2.3 The treatment and prognosis

**Figure 3:**
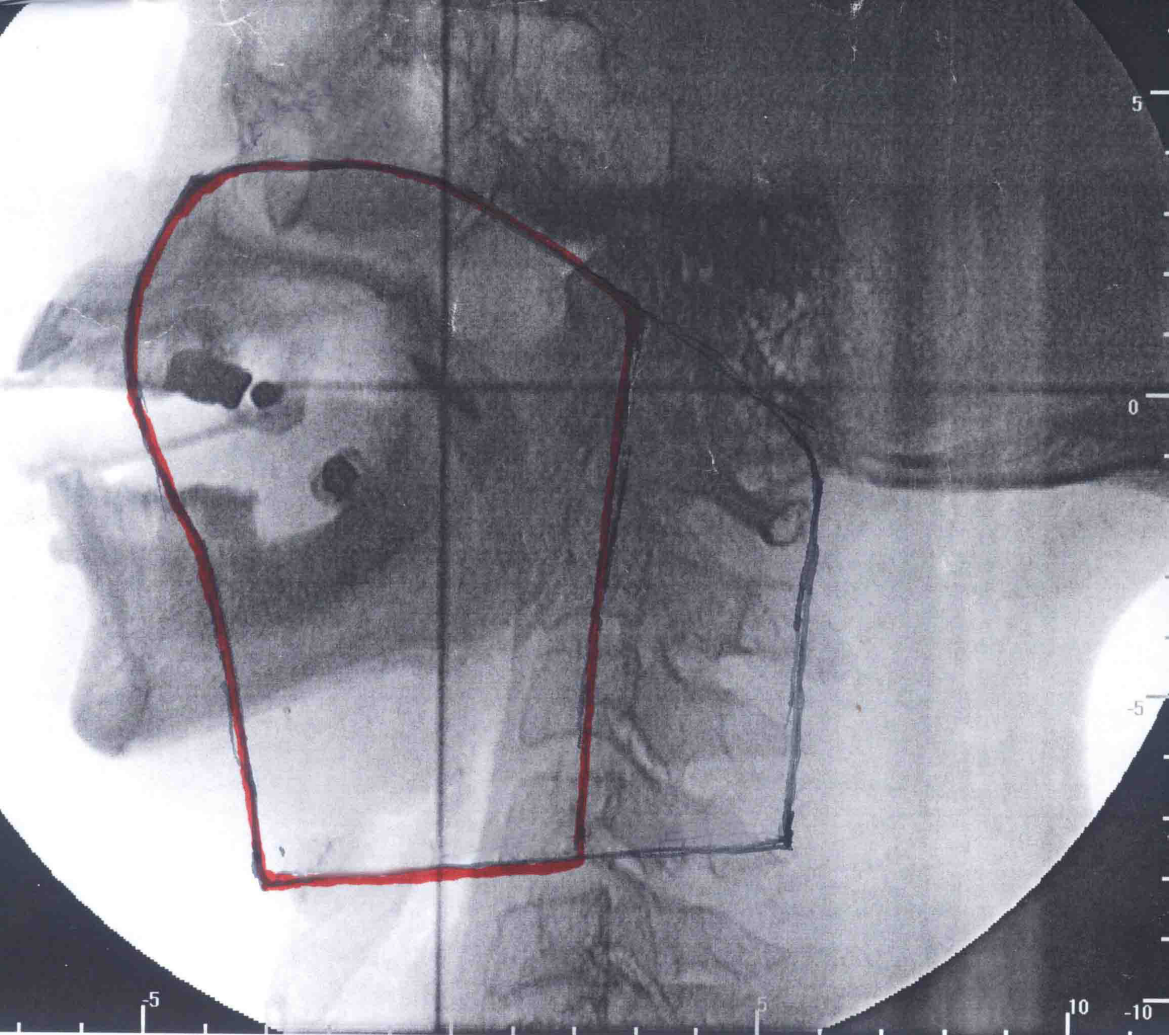
**The target region of radiotherapy included the tumor bed and regional lymph drainage area**.

Surgery is always the first choice of treatment for myoepithelial carcinoma, including extended tumor resection in the right palate and tumor exploratory in the maxillofacial/deep neck. Biomembrane was implanted and mass with incomplete capsule and intact bones were found. The whole layers of mucous membrane were incised at 1 cm away from the tumor edge by electric scalpel, and tumor was completely excised along the bone surface. After the surgery, both light microscope evaluation (routine HE staining) and immunohistochemical analysis using specific antibodies were carried out to confirm the pathological diagnosis of myoepithelial carcinoma. Focal external beam radiation-therapy was subsequently performed on the patient in the target regions of the tumor bed and regional lymph drainage area. The total tissue dose was 50Gy/25f (Figure 3), including the first target region (shown by black line in Figure 3) of 36Gy/18f and the second target region (the cervical spinal cord was sheltered, shown by red line in Figure 3) of 14Gy/7f, followed by 4 cycles of chemotherapy with Camptothecin. There was no evidence of local recurrence or distant metastasis in a period of 2-year follow-up.

## 3. Discussion

### 3.1 General concept of Myoepitheliomas

Myoepitheliomas are tumors arising from myoepithelial cells predominantly or exclusively. Myoepithelial cells are normally located between the epithelial cells and the basal lamina of acini and ducts of salivary glands, breast, and sweat glands of the skin. Thus the tumors mostly occur in the salivary glands, as well as in the breast, skin, and lung.

Myoepithelial carcinoma was described previously as malignant mixed tumor, however, exclusive myoepithelial differentiation makes it a distinctly separated tumor in pathology [4]. According to the WHO classification, myoepithelial carcinoma is referred to those lesions ''composed almost exclusively of tumor cells with myoepithelial differentiation''. Therefore, a tumor containing frequent true luminal differentiation should be excluded from the category of purely myoepithelial carcinoma'' [5].

Myoepitheliaomas and their malignant counterpart, myoepithelial carcinoma are distinct concepts from adenomyoepitheliomas [6]. Malignant adeno-myoepithelioma, adeno-myoepithelioma (combination of adenoma and myoepithelioma), epithelialmyoepithelial adenoma, and epithelial-myoepithelial carcinoma are the terms having exactly the same meaning [7]. However, when the myoepithelial cells were the major component of breast adenomyoepithelioma, it is usually called myoepithelial carcinoma.

There are no definite histological criteria for discriminating The benign and malignant myoepitheliomas can not be discriminated histologically. Indications of malignancy are based on features such as nuclear atypia, high mitotic rate and infiltrative growth into adjacent tissues. Currently, benign and malignant myoepitheliomas are differentiated by mitotic count, presence of invasive growth, cellular polymorphism, tumour necrosis, or their combination.

Compared with myoepitheliaomas, myoepithelial carcinoma shows aggressiveness and recurrence even after adequate therapy, and is disproportionately common in pediatric age group and has an aggressive clinical course [8]. Clinical presentation of myoepithelial carcinoma depends on its location. The growth pattern of myoepithelial carcinoma is generally multinodular, which comprise solid and sheet-like growths of tumor cells, with myxoid or collagenous/hyaline background frequently.

#### 3.1.1 Differences between Myoepitheliomas and Adenomyoepithelioma

Adenomyoepithelioma is characterized by simultaneous proliferation of epithelial and myoepithelial elements. According to the pathologists, other diagnostic terms with the same meaning have been used for adenomyoepithelioma of different organs, including adeno-myoepithelioma, malignant adeno-myoepithelioma, epithelialmyoepithelial adenoma, and epithelial-myoepithelial carcinoma [7]. But adenomyoepitheliomas are different concept from myoepitheliaomas and their malignant counterpart, myoepithelial carcinoma. Immunohistochemistry staining of adenomyoepithelioma demonstrates high expression of myoepithelial component such as S100 protein, Calponin, smooth muscle actin or/and GFAP, and negative expression of epithelial markers. The epithelial component usually reacts with cytokeratins and EMA, but is non-reactive with S100 and smooth muscle actin.

#### 3.1.2 Differences between Myoepitheliomas and Pleomorphic adenoma

As the most common minor salivary gland tumor (accounting for 40% of cases), pleomorphic adenoma shows epithelial/ductal cells in its tissues. These cells are small and cuboidal arranged in flat sheets or trabeculae that can undergo squamous, oncocytic, or sebaceous metaplasia. Myoepithelial cells are usually also present and can be spindled, or plasmacytoid and are found in clusters, singly, or within the chondromyxoid matrix. The presence of chondromyxoid matrix material is the most specific feature for making the correct diagnosis. There is abundant epithelial or myoepithelial cells with minimal stroma in cells of pleomorphic adenomas. The pleomorphic adenoma often show 8q12 and 12q13-q15 alterations cytogenetically, The relative lack of ducts and chondromyxoid stroma makes pleomorphic adenoma distinguished from myoepithelioma, which is composed almost exclusively of myoepithelial cells.

### 3.2 Histological features of Myoepitheliomas

In four major histological subtypes of myoepithelioma (epithelioid, spindle cell, plasmacytoid, and clear cell), the histological and ultrastructural features are well established, howwver, the cytologic criteria are obscure [9].

Myoepithelial cells are characterized by filaments on the basal site and pinocytotic vesicles at the ultrastructural level. Histologically, myoepithelial cells have varied cell morphology, including spindle cell, epithelioid, plasmacytoid and clear cell, or their combinations [10]. These four cell types were suggested to represent different stages in myoepithelial cell differentiation [11].

Histologically, most myoepitheliomas tumors are composed of cordal or nested plamacytoid, spindled cells or clear cells with various reticular architectures, and a myxoid, hyalinised or collagenous stroma. Some of these tumors show predominantly constant proliferation of spindled or plasmacytoid cells [12].

On the contrary, myoepithelial carcinoma is histologically characterized by a multilobulated architecture without duct formation and myoepithelial differentiation. Morphologically, the tumor cells are often spindle shaped, stellate, epithelioid, plasmacytoid (hyaline), and occasionally vacuolated with a signet-ring-like appearance. Solid sheet-like formations and trabecular or reticular patterns may form by tumor cells [13].

In the myoepithelial carcinoma of salivary gland, a malignant criteria proposed by Savera include the presence of seven or more mitoses per high-power field, tumor necrosis, and tumor infiltration of adjacent tissues, which was most predictive of malignant behavior [1].

### 3.3 Immunohistochemically features of Myoepitheliomas

Immunohistochemical analysis shows high expression of epithelial markers such as cytokeratin, epithelial membrane antigen (EMA), S-100 protein, and markers of smooth muscle origin such as smooth muscle actin and calponin on the tumor cells of myoepitheliomas. Current immunohistochemical criteria for the confirmation of myoepithelial differentiation are double positivity for both cytokeratins (pan CK or preferentially basal type CK) and one or more myoepithelial immunomarkers (i.e., S-100, calponin, p63, GFAP, maspin, and actins) [14-17].

The myoepithelial origin of myoepitheliomas was confirmed by simultaneous positive for actin, cytokeratin including CK-14 and Calponin along with S-100 in most reports. Moreover, EMA, glial fibrillary acidic protein and a variety of myogenic markers are also expressed in myoepitheliomas. However, it must be noted that these markers are not always positively expressed in the tumor cells and that negative staining does not necessarily exclude myoepithelial differentiation.

Immunohistochemical findings in our study are consistent with those of previous reports. In our cases, tumors immunoreactive for both cytokeratins and myoepithelial markers (S-100 and Calponin) confirmed the diagnosis of myoepithelial carcinoma and excluded the diagnosis of epithelial-myoepithelial carcinoma.

Some other kinds of tumors can also be excluded by Immunohistochemical findings. Histologically, spindle-like myoepithelial cellcarcinomas make close differential diagnoses with malignant melanoma (primary or metastatic). Both tumors is positive for S-100 protein, but melanoma usually does not stain positively for keratin, HMB-45 or myogenic markers. Epitheloid sarcoma usually stain positive only for EMA and keratins and negative for all other markers of myoepithelial differentiation, therefore, it can also be excluded [12]. Several other tumors can also be discriminated by immunohistochemistry features Foe instance, acinic cell carcinoma show positive to PASD; High-grade mucoepidermoid carcinoma show positive to mucicarmine, CEA, and muscle markers; Clear-cell oncocytoma of the salivary gland show positive to PTAH and mES mitochondrial antigen; Clear-cell sarcoma of soft tissue in the head and neck region show positive to melanoma-associated antigens, and show negative to CK; Metastatic clear-cell renal cell carcinoma show negative to intracytoplasmic lipids, S100 protein, and muscle markers [18].

### 3.4 Some genetic changes in myoepithelial carcinomas

#### 3.4.1 Genomic alterations in myoepithelial carcinomas

In general, myoepithelial carcinomas displayed slightly more chromosomal events than benign myoepithelioma. There are rarely genomic alterations in myoepithelial carcinomas especially in salivary and breast tissue.

However, Hedy reported that there are differential genomic alterations between myoepithelial carcinomas and benign myoepitheliomas of salivary gland. The most frequent gains of chromosomes in the benign tumors were at 22q11.1-q13.33 (40%) and 11q23.3 (38%). The recurrent gains of large genomic regions distinguish myoepithelial carcinomas from their benign counterparts. Chromosome copy number changes such as gains of whole chromosomes (chr. 8 in 27% and chr. 19 in 50%) or chromosome arms (20q in 32% and 22q in 50%) were frequently observed [19].

Laser capture microdissection and comparative genomic hybridization were performed by Jones to report genetic alterations in breast myoepithelial carcinomas [20]. The loss at 16q (3/10 cases), 17p (3/10), and 11q (2/10) were common alterations. The average chromosomal changes number of myoepithelial carcinoma of the breast (2.1, range 0-4) was lower that in unselected ductal carcinomas (8.6, range 3.6-13.8).

Magrini reported the cytogenetic features in the primary and metastatic myoepithelial carcinomas of salivary gland and showed a composite karyotype in the primary tumour: 45~46, XY, +3[cp3]/44~45, XY, -17[cp4]/46, XY [5]. The tumor cells from metastatic lymph-node was near-triploid and showed a complex karyotype [21].

#### 3.4.2 Changes of tumor suppressor gene

Both benign and malignant myoepithelial tumours of the salivary glands show deregulated expression in p16INK4a and p53 pathway members [22]. Benign tumour cells showed a higher expression of p16INK4a pathway members (p16INK4a, E2F1 and cyclin D1) compared with normal salivary gland. Furthermore, malignant tumours expressed p53 and EZH2 at a higher level. Recurrent tumors displayed more p53 positive tumour cells than primary tumors. The clear cell type of benign tumours had the highest proliferation fraction, and the plasmacytoid cell type showed a higher percentage of EZH2 positive cells. This indicates additional inactivation of p53 is needed in neoplastic transformation and aggressive tumour growth of myoepithelial carcinomas.

### 3.5 Treatment and Prognosis

Complete excision is the preferred treatment method for myoepithelioma [1]. For myoepithelial carcinoma, complete excision with tumor-free margin remains the first choice of treatment, in spite of the possibilities of local recurrence and distant metastasis. Local radiation therapy and chemotherapy are also needed for myoepithelial carcinoma.

Recurrence is rare for benign myoepithelial tumors, while the overall prognosis of myoepithelial carcinoma is poor. Several studies reported aggressive clinical behaviors for myoepithelial carcinoma, and the average metastatic rate was 47% and the mortality rate was 29% after a mean of 32 months.. Recurrence and metastasis are more common in children than in adult even with a negative excision margin [8]. Therefore, Yu suggested myoepithelial carcinomas of the salivary gland should be classified as high-grade malignancies [23].

In conclusion, we reported a very rare case of myoepithelial carcinoma from palate. We offer a diagnostic reference for classifying myoepithelial carcinoma based on the pathological characteristics. This report also provides the summary of the previous literature about myoepitheliomas and myoepithelial carcinoma. The concept, histological feature, immunohistochemistry feature, genetic changes, treatment options and prognosis of myoepithelial carcinoma are further discussed.

## Consent

Written informed consent was obtained from the patient for publication of this Case report and any accompanying images. A copy of the written consent is available for review by the Editor-in-Chief of this journal

## List of abbreviations

CK: cytokeratin; EMA: epithelial membrane antigen; GFAP: glial fibrillary acidic protein; HMB45: human melanoma black45.

## Competing interests

The authors declare that they have no competing interests.

## Authors' contributions

These authors contributed equally to this work.

## Funding

This manuscript is supported by the National Natural Science Foundation of China (30973175, Juan Ren), Scientific Research Foundation for Returning Scholars of Education Ministry of China (0601-18920006, Juan Ren), Scientific and Technological Research Foundation of Shaanxi Province (2007K09-09, Juan Ren), and the Clinical Research Fundation of First Hospital of Xi'an Jiao Tong University of China (Juan Ren).
